# Endoplasmic Reticulum Calcium Regulates the Retrotranslocation of *Trypanosoma Cruzi* Calreticulin to the Cytosol

**DOI:** 10.1371/journal.pone.0013141

**Published:** 2010-10-05

**Authors:** Carlos A. Labriola, Ianina L. Conte, Máximo López Medus, Armando J. Parodi, Julio J. Caramelo

**Affiliations:** 1 Laboratory of Glycobiology, Fundación Instituto Leloir and Instituto de Investigaciones Bioquímicas de Buenos Aires, Buenos Aires, Argentina; 2 Laboratory of Structural Cell Biology, Fundación Instituto Leloir and Instituto de Investigaciones Bioquímicas de Buenos Aires, Buenos Aires, Argentina; 3 Department of Biological Chemistry, School of Sciences, University of Buenos Aires, Buenos Aires, Argentina; Universidade Federal de Minas Gerais, Brazil

## Abstract

For most secretory pathway proteins, crossing the endoplasmic reticulum (ER) membrane is an irreversible process. However, in some cases this flow can be reversed. For instance, misfolded proteins retained in the ER are retrotranslocated to the cytosol to be degraded by the proteasome. This mechanism, known as ER associated degradation (ERAD), is exploited by several bacterial toxins to gain access to the cytosol. Interestingly, some ER resident proteins can also be detected in the cytosol or nucleus, calreticulin (CRT) being the most studied. Here we show that in *Trypanosoma cruzi* a minor fraction of CRT localized to the cytosol. ER calcium depletion, but not increasing cytosolic calcium, triggered the retrotranslocation of CRT in a relatively short period of time. Cytosolic CRT was subsequently degraded by the proteasome. Interestingly, the single disulfide bridge of CRT is reduced when the protein is located in the cytosol. The effect exerted by ER calcium was strictly dependent on the C-terminal domain (CRT-C), since a CRT lacking it was totally retained in the ER, whereas the localization of an unrelated protein fused to CRT-C mirrored that of endogenous CRT. This finding expands the regulatory mechanisms of protein sorting and may represent a new crossroad between diverse physiological processes.

## Introduction

Nearly one third of newly synthesized eukaryotic proteins are targeted to the secretory pathway. After entering the endoplasmic reticulum (ER) either post- or cotranslationally, most proteins are glycosylated, disulfide bridges are formed, and tertiary and quaternary structures acquisition is usually accomplished. At this stage, properly folded proteins leave the ER and travel to their final destination. The initial cross of the ER membrane is commonly a unidirectional process, although in some cases this flow can be reversed. For instance, proteins unable to acquire a stable fold are first retained in the ER by the folding quality control machinery to be next retrotranslocated to the cytosol for proteasomal degradation [1]. This process, known as ER associated degradation (ERAD), is exploited by some bacterial toxins to gain access to the cytosol [2]. These proteins only use the retrotranslocation capabilities of the ERAD machinery and once in the cytosol avoid being degraded. This illustrates that retrotranslocation does not necessarily imply an immediate degradation. Similarly to *bona fide* ERAD substrates, those bacterial toxins usually display low conformational stability, pointing to a similar recognition mechanism by the ERAD machinery [3], [4]. In addition, some ER resident proteins such as glucosidase II β (GIIβ) subunit, ERp57 (a member of the protein disulfide isomerase family) and calreticulin (CRT) can also be found in the cytoplasm and nucleus. The mechanism behind this anomalous targeting is obscure.

CRT is 46 kDa abundant ER resident protein that fulfils at least two basic functions [5]. Firstly, due to its capacity to bind monoglucosylated high mannose glycans, CRT is a central component of the glycoprotein folding quality control system. Secondly, CRT can bind high amounts of calcium (∼20 ions/protein) with low affinity (Kd ∼2 mM), being one of the main ER calcium buffers. CRT is formed by three structural domains. The N-terminal domain (residues 1–173, numbered according the sequence of mature *Trypanosoma cruzi* CRT, TcCRT) bears the sugar binding site. This domain is predicted to show a globular fold composed by two antiparallel beta sheets. The proline rich or P-domain (residues 174–284) is an extended hairpin that protrudes from the N-terminal domain. It participates in glycoprotein binding and interacts with ERp57, which collaborates in the folding maturation of glycoproteins bound to CRT [6]. Finally, the C-terminal domain (CRT-C) (residues 285–380) is highly enriched in negatively charged residues and is responsible for the calcium buffering activity of the protein. Although CRT is a typical ER resident protein, it has been found in several other locations including the cytosol, nucleus [7], [8], secretory granules [9], [10], the outer side of the plasma membrane [11], [12] and the extracellular space [13]. These alternative locations are linked with diverse biological roles. For instance, secreted CRT is involved in the modulation of the immunogenic response towards dying cancer cells [14]. In addition, topically applied CRT accelerates wound healing in a porcine model [15], [16]. On the other hand, cytosolic CRT regulates cell adhesion through its interaction with the cytosolic tail of integrin alpha subunit [17] and also mediates the nuclear export of some steroid hormone receptors [7], [18], [19]. Cytosolic CRT also regulates the stability or translational rate of some RNAs, such as angiotensin receptor AT1 [20], rubella virus RNA [21], C/EBPbeta and C/EBPalfa [22], p21 [23] and glucose transporter-1 [24]. Although these observations strongly suggest that CRT can reach the cytosol and nucleus, there is a persistent controversy on this issue. A good argument in favor of cytosolic/nuclear CRT is the detection of postranslational modifications exclusively occurring on those sites. For instance, CRT has been found to be phosphorylated by src kinase [25] and PKC [26], [27], it also can be modified in Ser/Thr with O-GlcNAc [28] and arginylated CRT has been found in cytosolic stress granules [29].

There are currently two models to explain the presence of CRT in the cytosol. The first one postulates the CRT signal peptide is suboptimal. According to this model, CRT start to translocate into the ER during their synthesis, but after cleavage of the signal peptide some ribosomes detach from the ER membrane and the synthesis is completed in the cytosol in free ribosomes [30]. The second mechanism postulates that CRT retrotranslotates to the cytosol from the ER lumen [31], probably taking advantage of the ERAD machinery. These models are no mutually exclusive, and their relative contribution to the pool of cytosolic CRT may depend on the physiological situation of the cell.

*N*-glycosylation in *T. cruzi* (the causative agent of Chagas disease) starts, at variance with higher eukaryotic organisms, with the transfer of a high mannose glycan (Man_9_GlcNAc_2_) devoid of glucose residues [32]. This feature was crucial in the discovery of the enzyme UDP-Glc:glycoprotein glucosyltransferase (UGGT), a central player in the glycoprotein folding quality control systems. TcCRT shows structural features similar to those found in other species. It binds monoglucosylated glycans and it is involved in the retention of immature species in the ER [33]. In particular, TcCRT plays an important role in the maturation of cruzipain (CZ), an abundant lysosomal protease and the first identified endogenous UGGT substrate [34]. Interestingly, TcCRT induces in mice an immune response against the host protein, raising the possibility that chagasic cardiomyopathy may be the outcome of an autoimmune response triggered by the parasite [35]. Although TcCRT is located mainly in the ER, histochemical studies have found it also in the Golgi complex, reservosomes, flagellar pocket, cell surface, cytosol, nucleus and kinetoplast [36]. The mechanism behind this heterogeneous localization is unknown.

We recently found that the conformation of CRT-C is affected by calcium in a concentration range similar to that spanned by the cation in the ER under physiological fluctuations [37]. In the presence of micromolar calcium concentrations CRT-C adopts an extended, random-like conformation, while calcium concentrations on the millimolar range induces a more rigid and compact structure. This information, combined with the several reports of cytosolic/nuclear CRT and the current knowledge about ERAD, suggests a mechanism that may regulates CRT localization within the cell. According to this model, ER calcium depletion would trigger a conformational change on CRT-C toward a more disordered structure. In this state, CRT would be recognized by the ERAD machinery, which would retrotranslocate the protein to the cytosol. Here we study the effect of ER calcium concentrations on the retrotraslocation of TcCRT.

## Materials and Methods

### Ethics Statement

This study was carried out in strict accordance with the recommendations in the Guide for the Care and Use of Laboratory Animals of the National Institutes of Health. The protocol was approved by the Institutional Committee on the Care and Use of Laboratory Animal of the Fundación Instituto Leloir (Permit Number: 2009/08/21JC). Blood extraction was performed under ketamine and xylasine anesthesia, and all efforts were made to minimize suffering.

### Reagents

Calcium ionophore A23187, cyclopiazonic acid (CPA), cycloheximide, proteasome inhibitor MG132, geneticin (G-418), trans-epoxysuccinyl-l-leucylamido (4-guanidino) butane (E-64), iodoacetamide, digitonin and Streptavidin-HRP were from Sigma. Anti-hemagglutinin (HA) and anti-green fluorescent protein (GFP) antibodies and 4-acetamido-4′-maleimidylstilbene-2,2′-disulfonic acid (AMS) were purchased from Invitrogen. Sulfo N-hydroxysulfosuccinimidyl biotin (sulfo-NHS-biotin) was purchased from Pierce. Antibodies anti-glutamate dehydrogenase (GDH), anti-CZ and anti-TcCRT were generated in rabbits from proteins either purified from the parasite (first two) or expressed in *E. coli* (last one).

### Cells and culture media

*T. cruzi* CL Brener epimastigotes were grown as described before [38]. *Escherichia coli* DH5α was used for cloning experiments. Bacteria were grown in Luria-Bertani medium, 0.5% NaCl, 1% tryptone (Difco), 0.5% yeast extract (Difco) and 100 µg/ml ampicillin if necessary.

### Genomic constructions

All constructions were performed in bacteria using pBluescript as vector. They were then subcloned in the trypanosomatid expression vector pTEX. The tetrapeptide KEDL-stop and the HA tag were generated by annealing of the corresponding primers and refilled with T4 polymerase (primers used are listed in Table S1). A pBluescript:HA-KEDL-stop was generated and TcCRT containing (bases 1–1179) or lacking (bases 1–1041) TcCRT-C was cloned upstream of HA. The sequence corresponding to TcCRT-C (bases 1040–1179) were inserted upstream of those of KEDL-stop in pBluescript:KEDL-stop. The GFP sequence was then inserted upstream of those corresponding to TcCRT-C or of KEDL-stop in the vector coding for GFP but lacking TcCRT-C. Finally, the TcCRT signal peptide was inserted to both constructions (GFP with or without TcCRT-C) by annealing and refilling with the corresponding primers.

### Cell disruption by freezing and thawing

The procedure employed was that described and characterized in Labriola *et al*, 1995. Briefly, cells were twice washed with 0.25 M sucrose, 5 mM KCI and the pellet obtained upon a low speed centrifugation was kept frozen at −20°C for 48 h, after which cells were thawed at 4°C and resuspended in 50 mM Tris-HCl, pH 7.6, 0.15 M NaCl, 1 µM E-64. The suspension was centrifuged for 10 min at 15,000× *g*. The supernatant was then removed (cytosolic fraction) and the pellet was resuspended in the above mentioned buffer supplemented with 1% NP-40. The suspension was centrifuged as above and the supernatant was removed (ER fraction).

### Parasite transfection and selection

CL Brener clone (4.10^7^ cells) epimastigotes in exponential growth phase were harvested, washed with phosphate buffered-saline (PBS), and resuspended in the same solution supplemented with 0.5 mM MgCl_2_ and 0.1 mM CaCl_2_. Parasites were mixed with 100 µg of the plasmid containing one of the constructions. The mixture, in a cuvette (0.4-cm electrode GenePulser, Bio-Rad) was subjected to electroporation (one pulse at 0.4 kV and 500 µF) in an ElectroCell Manipulator (BTX Electroporation System). Parasites were then incubated at room temperature for 10 min, diluted with 5 ml of BHT medium with 10% complete serum, and incubated for 48 h. G-418 was then added (250 µg/ml) and resistant parasites were selected over a 2 month period.

### Digitonin treatment

*T. cruzi* CL Brener clone epimastigotes were treated with digitonin (0–1 mg/ml) in Eppendorf microcentrifuge tubes as previously described [39]. The soluble and particulate fractions were separated by centrifugation at 20,800× *g* for 2 min at 4°C. GDH (cytosolic marker), CZ (lysosomal marker) and CRT (ER marker) were detected in the soluble fractions by Western blot using the appropriate antibodies.

### Labeling of surface proteins

Parasites were incubated with CPA for 20 min at 37°C and were centrifuged at low speed for 5 min at 4°C. Next, parasites were washed twice with PBS and resuspended in the same buffer. The sample was divided in two halves and one half was lysed by adding 1% NP-40. Amino groups of surface (non-lysed sample) or total proteins (detergent-treated sample) were labeled for 20 min at 4°C with 1 mM sulfo-NHS-Biotin. Excess reagent was quenched by adding 5 mM lysine for 30 min. CRT was immunoprecipitated and biotinylated CRT was detected by Western blot using Streptavidin-HRP. As a control, this procedure was also employed to label CRT present in subcellular fractions generated by freezing and thawing.

### Analysis of TcCRT disulfide bridge

To analyze the presence of a disulfide bridge in TcCRT, the ER and cytosolic fractions (see above cell disruption by freezing and thawing) were treated with 5 mM iodoacetamide for 5 min at room temperature to block free Cys residues. Next, the protein soluble fractions were reduced with 10 mM DTT in the presence of 8 M urea for 30 min at 37°C and treated with 30 mM AMS for 5 min at room temperature. Samples were then run on SDS-PAGE and analyzed by Western blot using anti-TcCRT antiserum.

## Results

### ER calcium depletion induces CRT retrotranslocation

**Figure 1:**
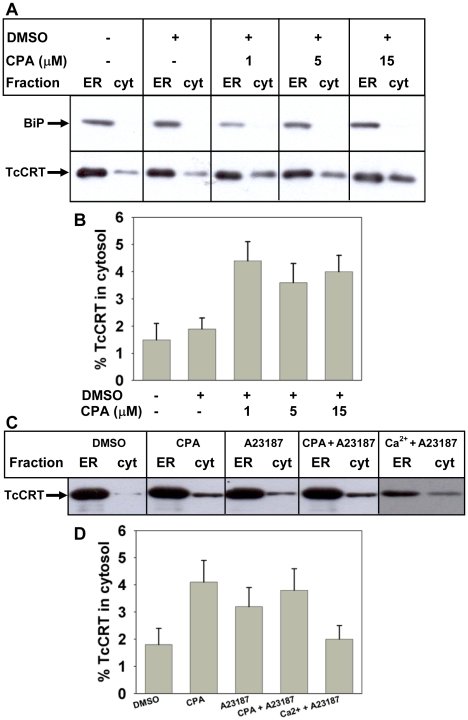
ER calcium mobilization induces TcCRT retrotranslocation. (**A**) Cellular distribution of TcCRT (lower gel) and BiP (upper gel) with no treatment or with the addition of DMSO and CPA as indicated. (**B**) Relative amount of cytosolic TcCRT with respect of total TcCRT of bands shown in (A). (**C**) Effect of the calcium ionophore A23187, CPA and/or external added calcium on the cellular distribution of TcCRT. (**D**) Quantification of the amount of cytosolic TcCRT with respect of total TcCRT of bands shown in (C). In B and C bars represent the standard error of triplicate measurements. In all cases the ER containing fractions were diluted ten fold.

As a first approach to study the intracellular localization of TcCRT we separated the ER from the cytosol plus lysosomal contents by freezing the parasites at −20°C, and thawing the sample at 4°C followed by centrifugation for 10 min at 15,000× *g*. This first supernatant contained the cytosolic plus lysosomal contents. The pellet was then resuspended in detergent and centrifuged. The second supernatant contained the lumenal ER content. This separation method has been characterized before [40]. Western blots of BiP and of an ER-tagged GFP (ER-GFP) showed that neither of them was found in the cytosolic plus lysosomal fractions (Figure 1A). In addition, a cytosolic version of GFP was completely absent from the ER fraction. These observations illustrate the efficiency of the cellular fractionation procedure. In contrast, the presence of TcCRT was evident in both fractions (Figure 1A). Cytosolic TcCRT accounted for about 2% of the total protein (Figure 1B). To evaluate whether ER calcium levels could influence TcCRT localization, we studied its variation resulting from ER calcium mobilization produced by the inhibition of the ER SERCA pump by CPA. This inhibitor was used instead of thapsigargin because the SERCA pump of the parasite is not inhibited by the latter [41]. Incubation with 1 µM CPA for 20 min promoted a ∼2 fold increment in the amount of cytosolic TcCRT. A similar ratio was obtained when using 5 µM or 15 µM inhibitor concentration (Figure 1B), suggesting that maximum inhibition was already reached at 1 µM. This last condition was routinely used in subsequent experiments. In addition, parasites were treated for 20 min with 10 µM of the calcium ionophore A23187 (Figure 1C and D). The amounts of cytosolic TcCRT using this drug alone or combined with 1 µM CPA were similar to that resulting from the use of only the last one. SERCA inhibition diminishes ER calcium levels with a concomitant increment of cytosolic calcium concentration. To analyze which of both phenomena (decrease of ER calcium or its cytosolic increase) triggered TcCRT relocalization, parasites were incubated with 5 mM CaCl_2_ and 10 µM A23187. This treatment promotes an increment of cytosolic calcium without major effects on its ER concentration. Under this condition the amount of cytosolic TcCRT was no altered compared with that of untreated parasites (Figure 1C and D). It may be concluded, therefore, that TcCRT relocalization was triggered solely by a decrease in ER calcium levels.

**Figure 2:**
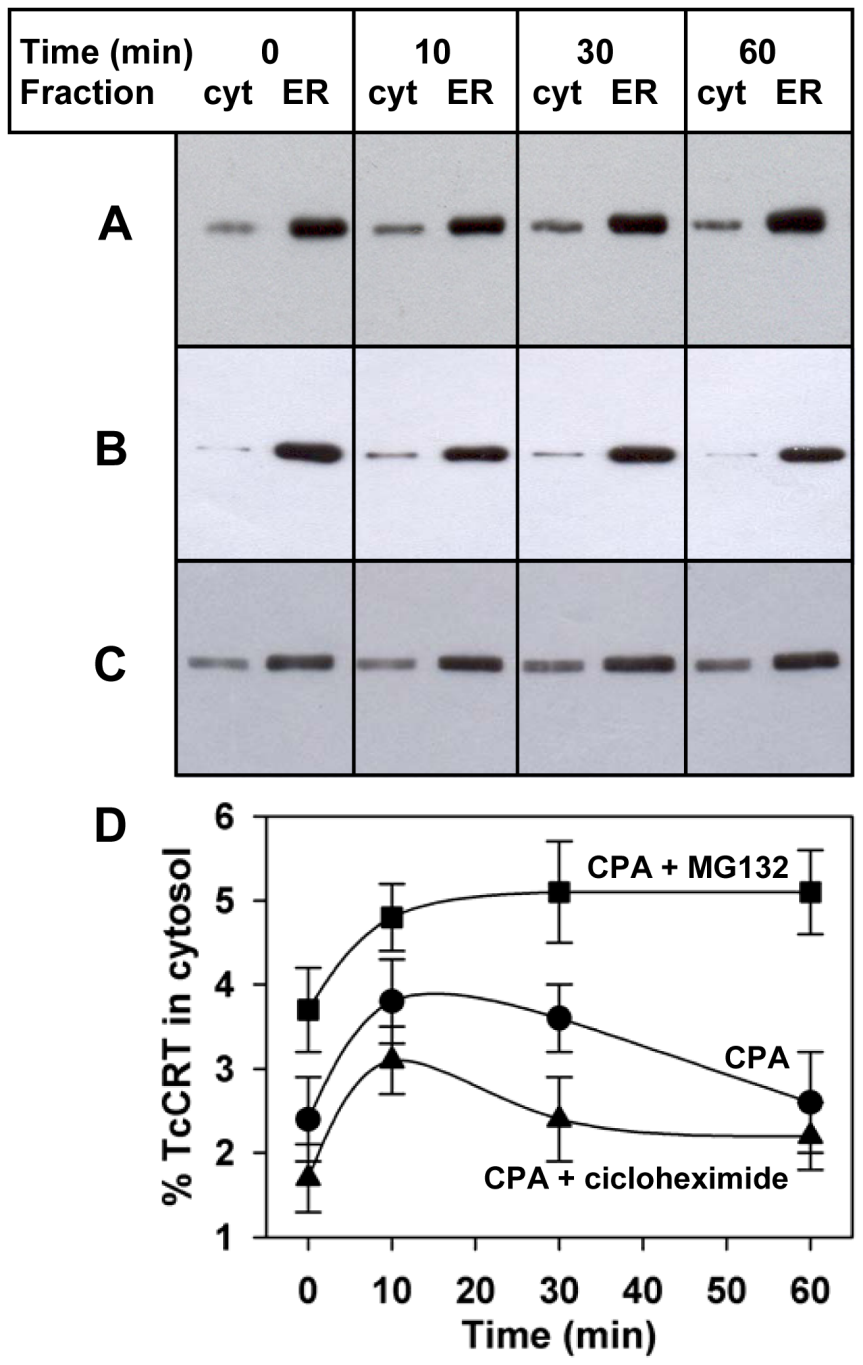
Retrotranslocated TcCRT is degraded by the proteasome. Cellular fractionation at different time points upon 1 µM CPA addition alone (**A**), or with a previous incubation for 1 h with (**B**) 1 mM cycloheximide or (**C**) 10 µM MG132. (**D**) Quantification of the fraction of cytosolic fraction of TcCRT shown in gels A–C. Bars represent the standard error of triplicate measurements. In all cases the ER containing fractions were diluted ten fold.

To analyze the kinetics TcCRT translocation as a result SERCA inhibition, cells were incubated in the presence of CPA and harvested at different time points. The increment in cytosolic CRT observed was relatively rapid, reaching a maximum after ∼20 min (Figure 2A and D). To address if the increase of cytosolic CRT was due to retrotranslocation from the ER or from a faulty *de novo* protein synthesis, we treated the parasites with cycloheximide before inhibiting the SERCA. Similar changes of cytosolic TcCRT were observed in the presence or absence of the protein synthesis inhibitor (Figure 2B and D), thus arguing in favor of the retrotranslocation hypothesis. Lower cytosolic TcCRT levels were observed in the presence of cycloheximide, thus suggesting that TcCRT was being degraded in the cytosol.

### Cytosolic TcCRT is degraded by the proteasome

The amount of cytosolic TcCRT reached a maximum ∼20 min after ER calcium depletion and diminished to resting levels within the next 60 min. This behavior indicated that the retrotranslocated protein had been cleared from the cytosol. To study whether cytosolic TcCRT was degraded by the proteasome, retrotranslocation was stimulated by CPA in the presence of the proteasome inhibitor MG132. Under this condition cytosolic TcCRT increased after the addition of CPA and its level remained steadily high (Figure 2C and D), suggesting that TcCRT concentration in the cytosol might be regulated by proteasomal degradation.

### Calcium induced TcCRT retrotranslocation depends on its C-terminal domain

**Figure 3:**
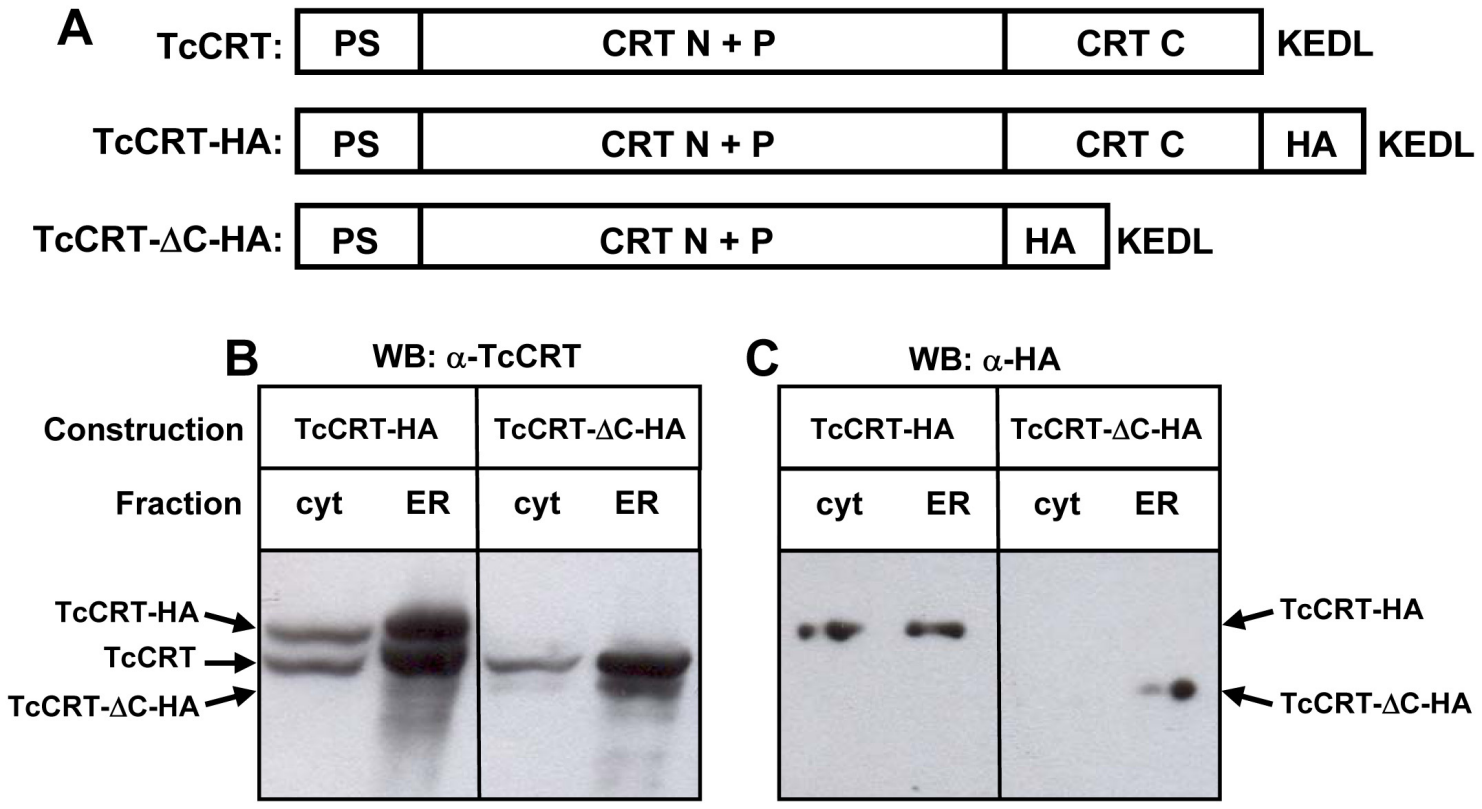
TcCRT devoid of its C-terminal domain is not retrotranslocated. (**A**) Constructions of TcCRT employed to address the effect of TcCRT-C. Cellular localization of constructs shown in (A) revealed by Western blot with (**B**) anti-TcCRT or (**C**) anti-HA antibodies. In (B) the endogenous protein is also visible. TcCRT-ΔC-HA refers to TcCRT lacking TcCRT-C. In all cases the ER containing fractions were diluted ten fold.

**Figure 4:**
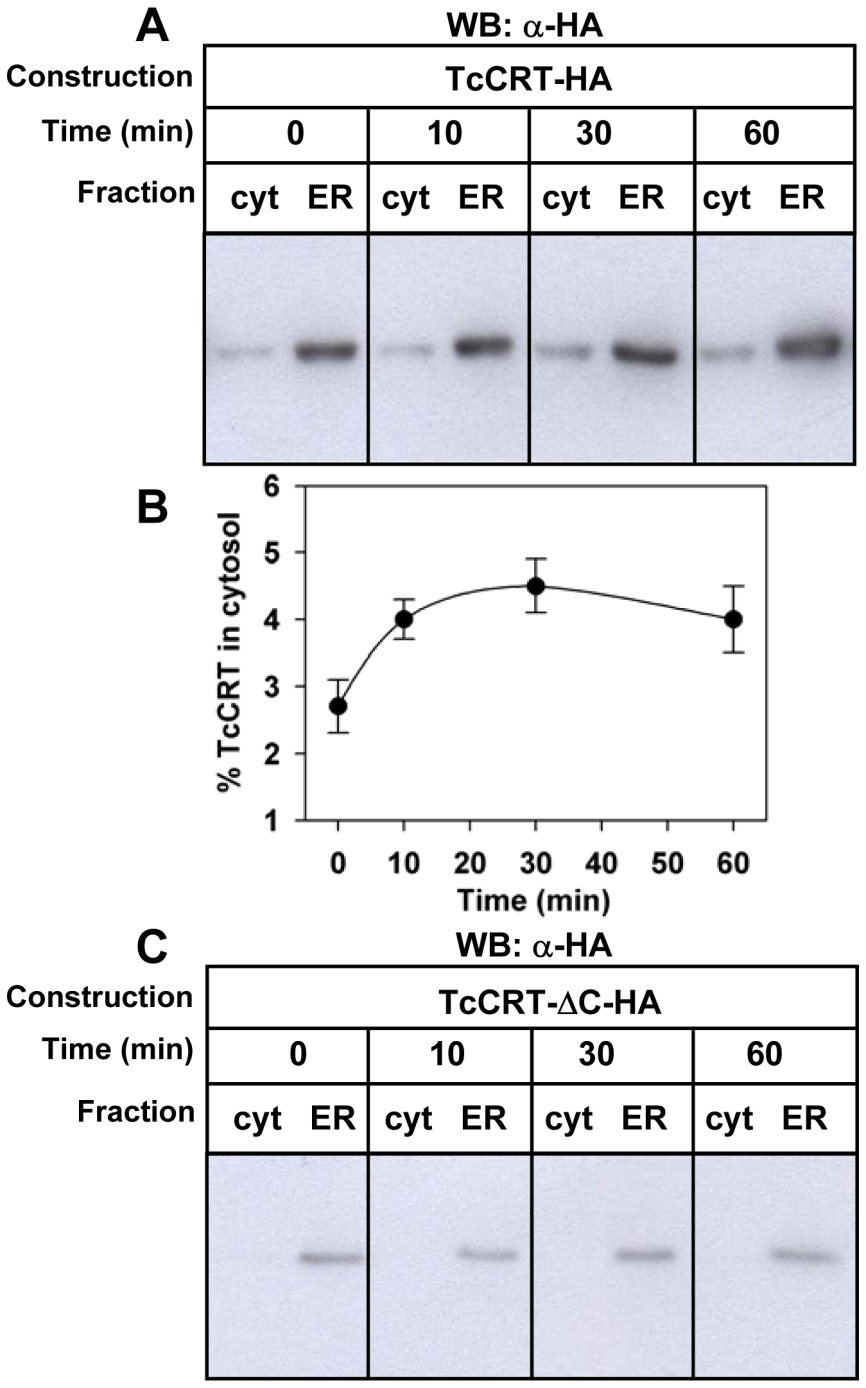
TcCRT devoid of its C-terminal domain is not retrotranslocated. Time course distribution of (**A**) TcCRT-HA or (**C**) TcCRT-ΔC-HA upon addition of 1 µM CPA revealed with anti-HA antibodies. (**B**) Relative amount of cytosolic TcCRT-HA of bands shown in (A). Bars represent the standard error of triplicate measurements. In all cases the ER containing fractions were diluted ten fold.

The presence of cytosolic CRT in mammalian cells depends on CRT-C [31]. To address this phenomenon in our system, we expressed an HA-tagged version of TcCRT with or without TcCRT-C (Figure 3A). The full length HA-tagged TcCRT appeared in both cytosolic and ER fractions, whereas the protein lacking the C-terminal domain was visible only in the last fraction, as revealed by Western blot using anti-TcCRT antibodies (Figure 3B). In both cases the endogenous TcCRT appeared in both compartments, with a distribution similar to that observed in non-transfected parasites. These results were confirmed using anti-HA antibodies (Figure 3C). In agreement with the previous experiments, cytosolic TcCRT was only observed with the whole protein, whereas the version lacking TcCRT-C was only visible in the ER fraction. Depletion of calcium from the ER by SERCA inhibition induced a time dependent increment of the cytosolic TcCRT of the full length HA-tagged protein (Figure 4A and B) with kinetic similar to that observed with the endogenous protein (Figure 1B), whereas the HA-tagged protein lacking TcCRT-C remained in the ER under all experimental conditions tested (Figure 4C).

**Figure 5:**
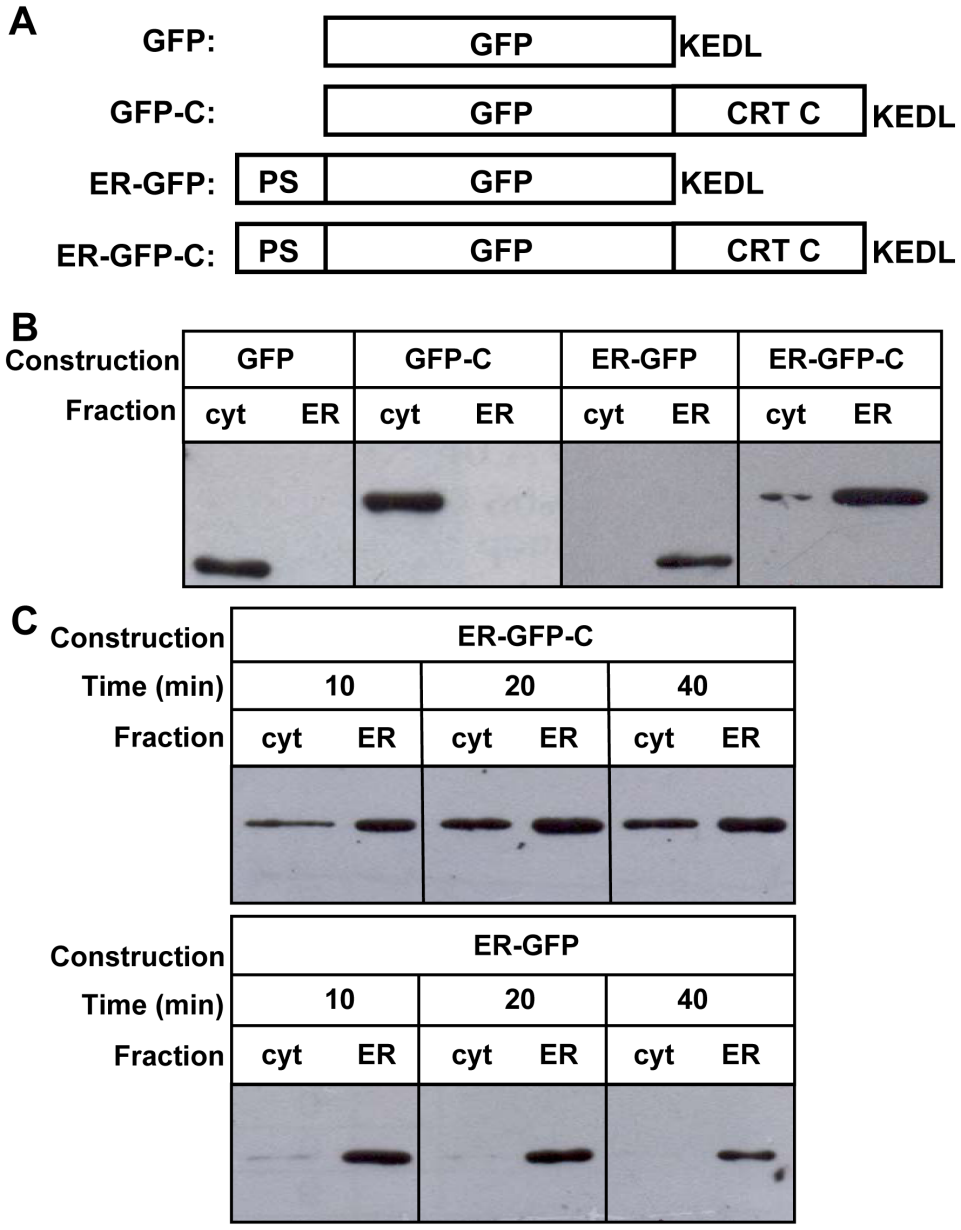
TcCRT C-terminal domain regulates the calcium response of retrotranslocation. (**A**) GFP-based constructs used to study the effect of TcCRT-C on protein localization. (**B**) Cellular localization of the GFP-based constructs. (**C**) Time course distribution of the ER-targeted GFP constructs with (upper gel) or without (lower gel) TcCRT-C after CPA addition. In (B) and (C) ER containing fractions were diluted 10 fold.

To further explore this issue, we expressed four constructs of GFP, with or without the addition of TcCRT-C, and targeted either to the cytosol or the ER. A C-terminal KEDL ER retention peptide was incorporated to all constructs (Figure 5A). Both GFPs lacking the signal peptide (i.e., GFP and GFP-C) appeared only in the cytosol, whereas both ER-targeted GFPs (i.e. ER-GFP and ER-GFP-C) were detected in the ER fraction (Figure 5B). In addition, ER-GFP-C was also detected in the cytosolic fraction with a distribution similar to that of the endogenous TcCRT (Figure 5B), thus demonstrating that the TcCRT-C is sufficient to sustain retrotranslocation. Moreover, depletion of ER calcium by CPA treatment induced an increment of the cytosolic pool of ER-GFP-C, whereas the cellular distribution of the ER-targeted GFP lacking TcCRT-C was not affected (Figure 5C). Taken together, these results showed that the retrotranslocation process and its response to ER calcium levels exclusively depend on TcCRT-C. We should mention that the original aim of using GFP was to follow the retrotranslocation process by confocal microscopy, but the minor amount of cytosolic GFP compared with that present in the ER precluded this approach.

**Figure 6:**
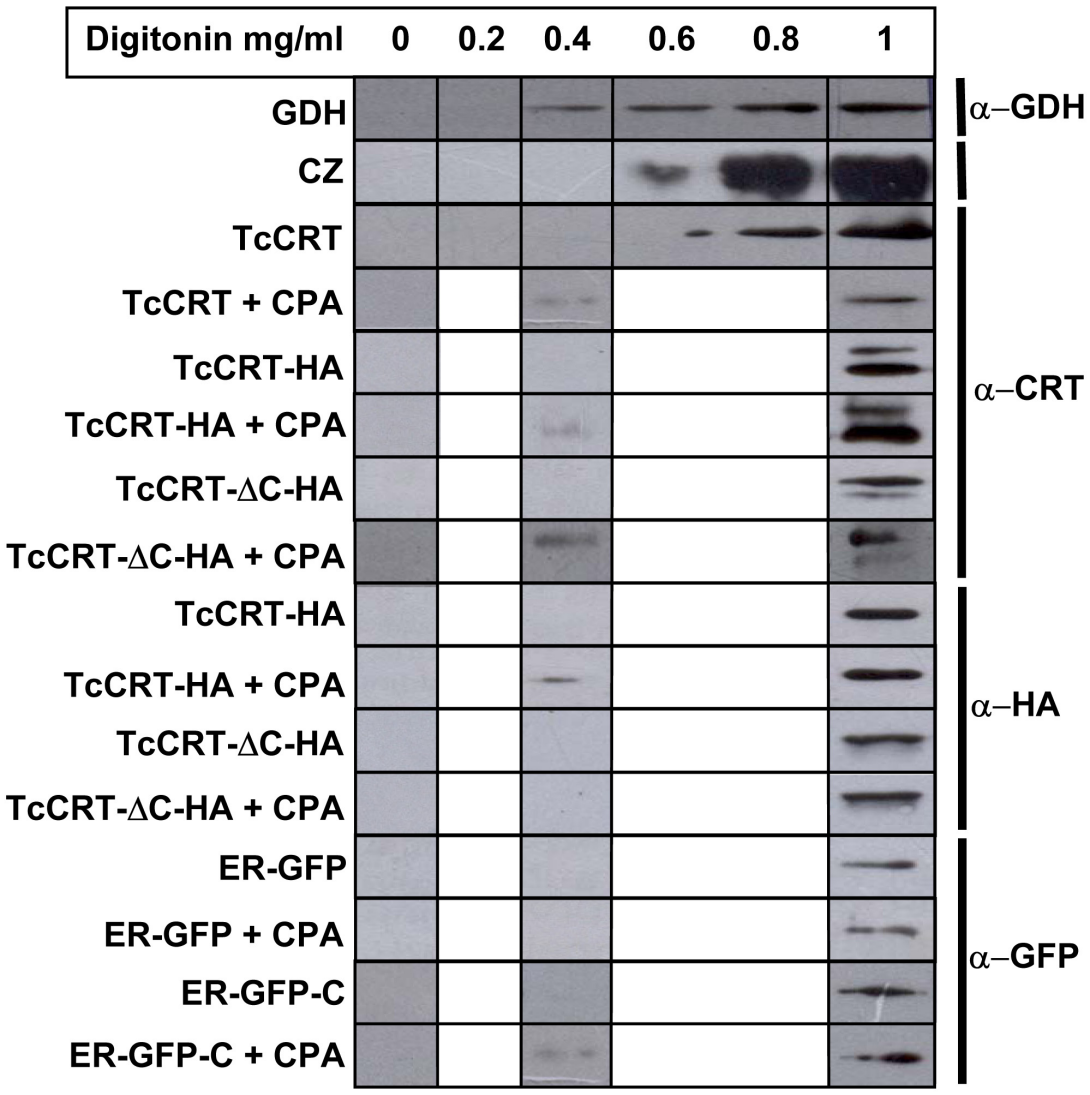
Protein localization analyzed using selective membrane permeabilization with digitonin. Detergent concentrations are indicated in the upper row. Left and right columns indicate the proteins analyzed and the antibodies employed for Western blots, respectively.

In order to corroborate these results we applied a second cellular fractionation procedure. The second strategy employed selectively permeabilizes the plasma membrane with digitonin. Release of the cytosolic marker GDH began when parasites were incubated with 0.4 mg/ml digitonin, whereas CZ, an abundant lysosomal marker, did so at 0.6 mg/ml (Figure 6). As permeabilization at 0.4 mg/ml of digitonin only allows the release of a minor fraction of total cytosolic proteins, endogenous TcCRT was visible in the cytosolic fraction only after treating parasites with CPA. Only the HA-tagged versions of TcCRT displaying the C-terminal domain appeared in the cytosolic fraction, and this was visible only when CPA was used. Identical results were obtained using either anti-HA or anti-TcCRT antibodies. Finally, constructs with GFP revealed a similar behavior, as the presence of cytosolic GFP after incubation with CPA was detected only when the protein was fused to TcCRT-C. Overall, these results fully agreed with those obtained using the alternative fractionation method. Furthermore, they show that TcCRT was translocated to the cytosol and not sorted to lysosomes upon CPA treatment.

**Figure 7:**
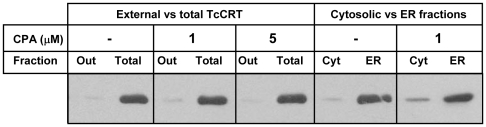
Effect of calcium mobilization on the amount of membrane-bound TcCRT. Intact, non-lysed (“out”) or lysed, detergent-treated (“total”) parasites were treated CPA and proteins were modified with sulfo-NHS-biotin. TcCRT was immunoprecipitated and revealed by western blot using Streptavidin-HRP. As a control, lysosomal and ER fractions were treated similarly.

It may be speculated that ER calcium mobilization might promote TcCRT secretion and that this phenomenon may explain, by cross contamination, the increase observed in cytosolic TcCRT. Although this suggestion is ruled out by the fact that parasites were washed twice with PBS before subcellular fractionation, it might be further speculated that an increased TcCRT secretion might in turn result in an increased TcCRT unspecific adherence to *T. cruzi* plasma membrane, which in turn would contaminate in some way the cytosolic fraction. To explore this possibility, parasites were labeled with sulfo-NHS-Biotin, a membrane impermeable reagent employed to biotinylate external proteins. The presence of biotinylated TcCRT was analized by immunoprecipitation of TcCRT followed by Western blot using Streptavidin-HRP. Only a very small proportion of TcCRT was biotinylated, probably due to a low fraction of dead, lysed parasites. More important, the amount of biotinylated TcCRT was not affected by CPA addition (Figure 7). Sulfo-NHS-biotin was also employed to asses the proportion of TcCRT present in the lysosomal and ER fractions. In this case, an increment of biotinylated TcCRT was clearly observed in the cytosolic fraction upon CPA treatment (Figure 7). This experiment shows that the increase of cytosolic TcCRT induced by CPA was a consequence of its relocalization to the cytosol and not of an increased association to the plasma membrane and a subsequent contamination of the cytosolic fraction.

### The disulfide bridge of cytosolic TcCRT is reduced

**Figure 8:**
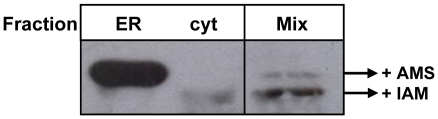
Oxidation state of TcCRT in the ER and cytosol. Samples were first alkylated with iodoacetamide (IAM), followed by reduction with DTT and alkylation with AMS. Rigth lane: mix of samples reduced with DTT and alkylated exclusively with either IAM or AMS.

Proteins that retrotranslocate from the ER to the cytosol must be previously unfolded, probably because they cross the ER membrane through a narrow pore. This implies that proteins displaying disulfide bridges must be previously reduced in the ER. TcCRT has a single disulfide bridge between Cys83 and Cys114. To analyze the presence of this disulfide bridge in TcCRT, the ER and cytosolic fractions were treated with iodoacetamide to block free Cys residues. Next, the soluble protein fractions were reduced with 10 mM DTT in the presence of 8 M urea and treated with 30 mM AMS. This reagent blocks free Cys and adds a molecular mass of approximately 500 Da to each modified residue, thus reducing the gel mobility of proteins displaying disulfide bridges. Clearly, TcCRT migration depended on its cellular localization, as the protein present in the ER showed a migration slower than that present in the cytosol (Figure 8). The higher mobility of cytosolic TcCRT could be due to proteolysis or deglycosylation, but the identical gel migration of cytosolic and ER samples observed in Figure 1 ruled out this possibility. This experiment shows that cytosolic TcCRT is reduced, whereas the protein present in the ER is oxidized and, furthermore, argues against the possibility that the former TcCRT could be a result of leakage of the ER protein during the fractionation procedure.

## Discussion

Although CRT resides mainly in the ER lumen, several reports have found it in other locations such as cytosol, nucleus, secretory granules, the outer side of the plasma membrane and the extracellular space. Accordingly, CRT has been associated with several biological functions, its roles as a lectin/chaperone in the folding of glycoproteins and as calcium buffer within the ER being the most prominent. Given the myriad of cellular responses mediated by calcium, many of the biological effects observed upon changes in CRT levels might originate from an imbalance of calcium homeostasis. For this reason, the biological relevance of cytosolic CRT is controversial.

Here we showed that a minor fraction of TcCRT is present in the cytosol. The presence of TcCRT in the cytosol may be a consequence of retrotranslocation or the result of an inefficient translocation during protein synthesis. The fact that TcCRT devoid of the C-terminal domain was absent from the cytosol favors the former interpretation. Aborted ER translocation would operate during the first steps of protein synthesis and the C-terminal-truncated TcCRT and the full length protein should be expected to behave similarly. Moreover, the fact that enhancement of cytosolic CRT content triggered by CPA treatment occurred even in the absence of protein synthesis also favors the idea that CRT is retrotranslocated from the ER lumen to the cytosol. We also found that the amount of protein located in the cytosol increased upon perturbing ER calcium levels, showing a relatively fast and transient response. Cytosolic TcCRT levels peaked about 15–20 min after ER calcium mobilization and the evidence suggests that the proteasome is involved in the subsequent clearance of CRT from the cytosol. As these observations were made with the endogenous protein we can rule out a mislocalization due to saturation of cellular sorting systems. We also found that a shorter version of TcCRT lacking the C-terminal domain was totally retained in the ER, whereas a non related protein such as GFP fused to this same domain mimicked TcCRT localization. These results show that ER calcium level regulates the C-terminal-dependent retrotranslocation of TcCRT. Cellular fractionation, selective membrane permeabilization and protein biotinylation methods yielded similar results.

Calcium concentration in the ER varies over two orders of magnitude, from approximately 5 mM when it is full to about 50–100 µM after the opening of the ER membrane calcium channels [42], [43]. Strikingly, CRT structure is marginally stable at physiological temperatures and CRT melting temperature diminishes from 46.8°C to 43.5°C when calcium concentration decreases from 1 mM to 10 µM [44]. Similar conditions facilitate the recognition by CRT of hydrophobic peptides [45]. In addition, we have recently found that the conformation of CRT-C is affected by calcium in a concentration range similar to that spanned in the ER during physiological fluctuations [37]. In the presence of millimolar calcium concentrations the domain adopts a rigid and compact structure whereas micromolar calcium concentrations induce an extended and random-like conformation. Although those results were obtained using rabbit CRT C-terminal domain, we suggest that similar conformational changes occur in TcCRT-C upon calcium concentration variations, as both the mammalian and protozoan C-terminal domains share similar sequence features. As bacterial toxins and the ERAD substrates usually display unstable conformations [3], [4], a feature that would favor their unfolding by the ERAD machinery, we speculate that TcCRT reaches the cytosol through the ERAD system. However, at variance with ERAD substrates and bacterial toxins, we propose that CRT retrotranslocation is modulated by structural modifications triggered by variations of ER calcium levels. We suggest a model in which ER calcium depletion would trigger a conformational change on CRT C-terminal domain toward a more disordered structure. In this state CRT would be recognized by the ERAD machinery, which would then retrotranslocate the protein to the cytosol. Interestingly, the single disulfide bridge of TcCRT is reduced when the protein is located in the cytosol, a likely consequence of its reduction in the ER previous to retrotranslocation. A member of the protein disulfide isomerase (PDI) family could be involved in this process. Indeed, PDI is required for the retrotranslocation of several ERAD substrates and toxins [46], [47]. Interestingly, CRT association *in vitro* with ERp57 and PDI is modulated by calcium. Whereas at high calcium concentration CRT associates with ERp57, lowering the ion concentration to 100 µM promotes the disassembly of this complex and the association of CRT with PDI [48]. Moreover, the calcium-regulated interaction between PDI and CRT depends on CRT-C. We are currently studying the association between PDI and CRT *in vivo*.

CRT is not the only ER resident protein found in the cytosol. This behavior is also observed for ERp57 and GIIβ. ERp57 binds Stat3 in the cytosol, thus inhibiting Stat3 DNA binding activity [49], although these results have been recently challenged [50]. ERp57 may also bind to Ref1 [51], a protein involved in DNA repair, or directly to DNA in the nucleus [52], [53]. On the other hand, GIIβ which plays an important role in the processing of *N*-glycans in the ER [54], has also been identified as a substrate of PKC [55], [56]. Since PKC activation is usually accompanied by ER calcium mobilization, it is likely that this signal could trigger the retrotranslocation of GIIβ, thus becoming accessible to PKC. In addition, it was recently found that GIIβ interacts with the cytosolic tails of IP3 receptor [57] and TRPV5 calcium channel [58], thus providing further evidence of its cytosolic localization. GIIβ and CRT share some structural features. Both proteins are marginally stable and present long stretches of negatively charged residues. Therefore, both proteins could undergo a similar retrotranslocation processes.

Whether the biological activities of cytosolic TcCRT are similar to those already described for its mammalian homologue is presently unknown. We are currently addressing whether ER calcium depletion also triggers CRT retrotranslocation in mammalian cells. Preliminary results indicate that this is the case. Which could be the biological relevance of this process? Nuclear export of glucocorticoid receptors mediated by CRT requires high calcium concentrations and is dependent on its C-terminal domain [59]. These experiments were performed with exogenously added CRT to semipermeabilized cells, a non-physiological situation. Nevertheless, these observations combined with the results presented here could be the basis for an interesting phenomenon, in which the cellular response to steroid hormones could be modulated by signals that generate inositol triphosphate. In a cell activated by a steroid hormone its receptor will be located in the nucleus. The arrival of a signal that mobilizes ER calcium would trigger an increment of cytosolic calcium and favor CRT retrotranslocation, thus fulfilling both known conditions for CRT-mediated nuclear receptor export. It has been recently suggested that nuclear export of androgen receptor is independent of CRT [60]. Given our observations, it would be interesting to study the effect of ER calcium mobilization on the fate of steroid-activated nuclear receptors. Finally, evidence presented here and previous results by others suggest that the regulation of CRT retrotranslocation by calcium could be a crossroad between diverse signaling pathways. This model could elucidate diverse and sometimes contradictory observations regarding this fascinating protein.

## Supporting Information

Table S1 Primers used for constructions
(0.02 MB PDF)
