# Supplementary material for: Endoplasmic Reticulum Calcium Regulates the Retrotranslocation of Trypanosoma Cruzi Calreticulin to the Cytosol
Source: PLoS One. 2010 Oct 5;5(10):e13141. doi: 10.1371/journal.pone.0013141 (PMC2950133; doi:10.1371/journal.pone.0013141)
Supplement: Table S1 — Primers used for constructions (0.02 MB PDF) [file pone.0013141.s001.pdf]

Primer	Sequence	Restriction sites	Purpose
**1**	5´-GACGAAAAGGGTGACCGTGATAAGGAG-3´		Generate KDEL. Used with primer **2** and T4 polymerase. The fragment generated was cloned in pBLUESCRIPT digested with Xho I-Hinc II
**2**	5´-CCG**CTCGAG**TTACAAATCCTCCTTATC-3´	**XhoI**	see primer **1**
**3**	5´CCC**AAGCTT**ATCTTTTACCCA 3´	**HindIII**	Amplification of 3 X HA tag from pSLF-72 vector. Used with primer **4**. The product was cloned in pBLUESCRITP:KDEL digested with Hind III-EcoR V
**4**	5´-CCGGGTCGA**GATATC**AGCGTA-3´	**EcoR V**	see primer **3**
**5**	5´-CCC**AAGCTT**TTCCAACTCTTCTTC-3´	**Hind III**	Amplification of CRT. Used with primer **7**. The fragment was cloned in pBLUESCRIPT:HA-KDEL digested with Hind III-EcoR I
**6**	5´-CCC**AAGCTT**TCCGCCTTCTTCAGG-3´	**Hind III**	Amplification of CRT-ΔC. Used with primer **7**. The fragment was cloned in pBLUESCRIPT:HA-KDEL digested with Hind III-EcoR I
**7**	5´-G**GAATTC**ATGCGTGCAGCA-3´	**EcoR I**	see primer **5**
**8**	5´-CCC**AAGCTT**TTCCAACTCTTCTTC-3´	**Hind III**	Amplification of CRT-C. Used with primer **9**. The product was cloned in pBLUESCRIPT:KEDL digested with EcoR V-Hind III
**9**	5´-ATCGGCCTGAAGAAAGCG-3´		see primer **8**
**10**	5´-G**GAATTC**ATGGTGAGCAAG-3´	**EcoR I**	Amplification of GFP. Used with primer **11**. The fragment was cloned in pBLUESCRIP: KEDL or pBLUESCRIPT:CRT-C-KDEL, both previously digested with EcoR I-EcoR V
**11**	5´-CTTGTACAGCTCGTCCATC-3´		see primer **10**
**12**	5´-GGCCTTGCGACACTGTCAGCCGTGCA CGGCACGGTGAGCAAGGGCGAGGAGC-3´		Introduction of signal peptide by partial annealing with templates pBLUESCRIPT:GFP-KEDL or pBLUESCRIPT:GFP-CRT-C-KEDL. Used with primer **13**
**13**	5´-CAAAAGGGCACAGAAAAAAATTGCTG CACGCATGAATTCCTGCAGCCCGGG-3´		see primer **12**
